# Transcriptomic Analysis Reveals Transcription Factors Related to Leaf Anthocyanin Biosynthesis in *Paeonia qiui*

**DOI:** 10.3390/molecules22122186

**Published:** 2017-12-08

**Authors:** Jianrang Luo, Jingjing Duan, Dan Huo, Qianqian Shi, Lixin Niu, Yanlong Zhang

**Affiliations:** 1 College of Landscape Architecture and Art, Northwest A&F University, Yangling 712100, Shaanxi, China; luojianrang@163.com (J.L.); duanjingjing@nwafu.edu.cn (J.D.); huodanstu@163.com (D.H.); shiqianqian2005@163.com (Q.S.); 2 National Engineering Research Center for Oil Peony, Yangling 712100, Shaanxi, China

**Keywords:** tree peony, transcriptome, anthocyanins, differentially-expressed genes (DEGs), transcription factors

## Abstract

*Paeonia qiui* is a wild species of tree peony. *P. qiui* has good ornamental value owing to its leaf color change in spring. So far, the molecular mechanism of leaf color change in *P. qiui* is unclear. This study analyzes the anthocyanin level and transcriptome of two different color stages in *P. qiui* leaf. The purplish-red leaf stage is rich in anthocyanin, while the green leaf has very low levels of anthocyanin. Transcriptome analysis reveals that 6678 differentially-expressed genes (DEGs) are up-regulated, and 14,667 are down-regulated in the purplish-red leaf. Among these DEGs, 40 MYB (v-myb avian myeloblastosis viral oncogene homolog) genes, 40 bHLH (MYC-like basic helix–loop–helix) genes, and 15 WD40 (WD-repeat protein) genes were found. Based on phylogenetic and alignment analysis with the deduced amino acid sequences with known transcription factors, Unigene0024459 (MYB1) is likely the R2R3-MYB that promotes anthocyanin biosynthesis; Unigene0050761 (MYB2) is likely the R2R3-MYB that represses anthocyanin biosynthesis; Unigene0005081 (bHLH1) and Unigene0006146 (WD40-1) are likely the bHLH and WD40 that participate in regulating anthocyanin biosynthesis. Additionally, quantitative RT-PCR results confirmed the transcriptome analyses for key genes.

## 1. Introduction

The spring leaf color change is one of the most remarkable events in nature, and has unique ornamental value. The beauty of the spring leaf color attracts many tourists to landscape architecture, and brings significant economic benefits. Tree peony (*Paeonia suffruticosa*) is a woody shrub of the genus *Paeonia* and family Paeoniaceae, and is a traditional ornamental plant [1]. There are nine wild species: *P. qiui*, *P. ostii*, *P. rockii*, *P. jishanensis*, *P. decomposita*, *P. delavayi*, *P. lutea*, *P. potanini*, and *P. ludlowii*. Among these wild species, the spring leaf color of *P. qiui* is the most striking. The leaf color of *P. qiui* is purplish-red before bloom (peonidin 3,5-di-*O*-glucoside is the main pigment), and the purplish-red of its leaf will fade gradually after bloom. The colors of the plant organs are mainly attributed to the accumulation of anthocyanins, a class of plant flavonoid metabolites [2,3,4]. The anthocyanin biosynthetic pathway has been well studied in plants. There are many enzymes that can catalyze anthocyanin synthesis, such as chalcone synthase (CHS), chalcone isomerase (CHI), dihydroflavonol 4-reductase (DFR), anthocyanidin synthase [2], etc. The enhanced expression of structural genes of anthocyanin biosynthesis accounts for increased levels of anthocyanin accumulation in plants directly [5]. However, these structural genes are usually regulated by transcription factors, including DNA-binding R2R3 MYB, MYC-like basic helix–loop–helix (bHLH), and WD40-repeat proteins [3,4,5,6,7].

In the absence of a complete genome sequence of tree peony, transcriptomic analysis is an effective method for gaining insights into differentially-expressed genes (DEGs) between differently-color leaves. Transcriptome sequencing of the different leaf color stages of *P. qiui* will provide useful insights into the molecular mechanisms of its leaf color changes in spring. To date, researchers mainly focused on floral flavonoid/anthocyanin component detection [8,9,10] and petal flavonoids/anthocyanin structural genes expression analysis in the tree peony [11,12,13,14]. However, the molecular mechanism of tree peony leaf color changes in spring is unclear.

In this study, the transcriptomic analysis was conducted from the red leaf stage and the green leaf stage in *P. qiui*. Candidate genes, including structural genes and transcription factors involved in leaf anthocyanin biosynthesis, are obtained by analyzing DEGs. The main objective is to reveal the molecular mechanisms on leaf color changes in spring, which will help people understand this remarkable event in nature.

## 2. Results

### 2.1. Anthocyanin Level in the Different Leaf Stages of P. qiui

**Figure 1:**
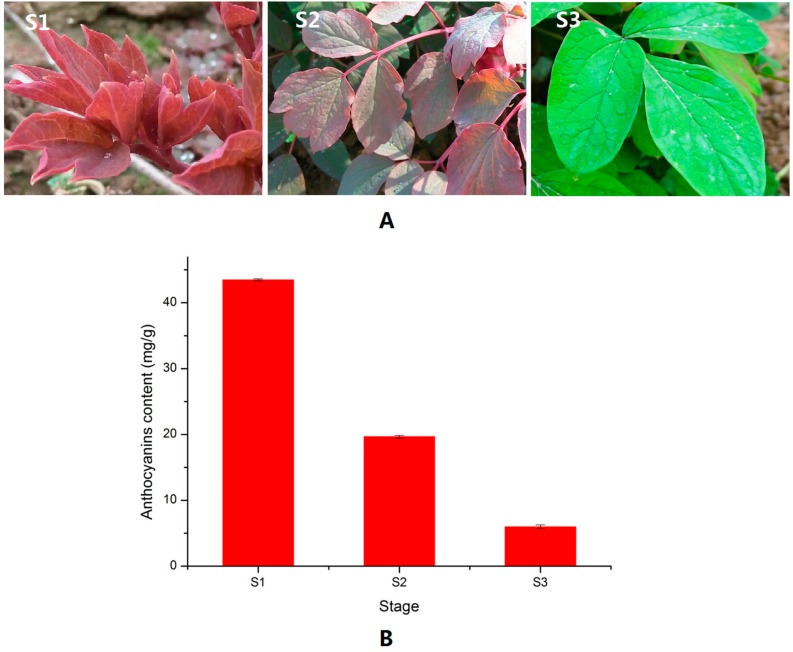
Images of *P. qiui* leaf and anthocyanin contents at three stages (S1: leaf does not spread fully; S2: leaf spread fully; and S3: flower just faded). (**A**) Images of *P. qiui* leaf at three different stages; and (**B**) contents of anthocyanin in three different leaf stages.

The leaf color of *P. qiui* in spring is usually purplish-red before bloom; then its color fades gradually (Figure 1A). The leaf color of *P. qiui* is purplish-red at S1 (leaf does not spread fully), then changes to half purplish-red and half green at S2 (leaf spread fully) and, finally, fully green at S3 (flower just faded). As shown in Figure 1B, the anthocyanin contents decreased from S1 to S3. This indicated that the red color fading of *P. qiui* was mainly caused by anthocyanin content changes.

### 2.2. Library Construction, De Novo Assembly, and Gene Annotation

To understand the molecular mechanisms of leaf color change, the leaves of S1 and S3 in *P. qiui* were used to build two libraries for high-throughput sequencing. After removal of adaptor sequences, ambiguous reads, and low-quality reads, 59,927,621 and 56,802,321 high quality clean reads comprising 8.82 Gb and 8.36 Gb nucleotides with Q20 > 98% were generated from S1 and S3 transcriptome sequencing, respectively. In total, 59,541 unigenes with an N50 of 1209 were generated by Trinity software (Broad Institute, Cambridge, MA, USA) [15]. The minimum length, maximum length, and average length of these unigenes were 200 nt, 11,919 nt, and 808 nt, respectively. The size distributions of these unigenes are shown in Supplementary Figure S1.

**Figure 2:**
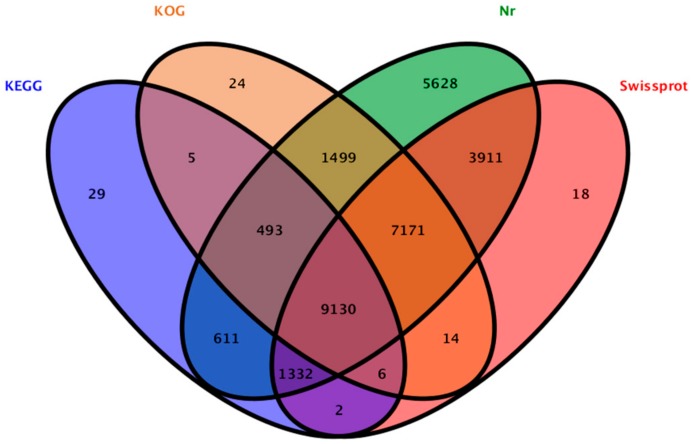
Outcome of the homology search of *P. qiui* unigenes.

A total of 29,837 unigenes (50.11% of all 59,541 cleaned unigenes) were annotated based on BLASTx (http://www.ncbi.nlm.nih.gov/BLAST/) (*E*-value < 1 × 10^−5^) searches of four public databases. Among them, 29,775 unigenes, 21,584 unigenes, 18,342 unigenes, and 11,608 unigenes could be annotated to the NCBI-nr database (http://www.ncbi.nlm.nih.gov), Swiss-Prot (http://www.expasy.ch/sprot), and COG/KOG databases (http://www.ncbi.nlm.nih.gov/COG), respectively (Figure 2).

### 2.3. Identification of DEGs Involved in Flavonoid/Anthocyanin Biosynthesis

The threshold false discovery rate (FDR) < −0.05 and an absolute value of log2 ratio > 1 were used to judge the significance of the DEGs in S1 versus S3. There were 21,445 DEGs between the S1 and S3 libraries, in which, 6678 unigenes were significantly up-regulated and 14,667 unigenes were down-regulated. Among those differentially expressed genes with a KEGG pathway annotation (http://www.genome.jp/kegg), 2333 DEGs were mapped to 126 pathways (Supplementary Table S2).

Through annotation in the KEGG database, 25 DEGs were predicted to participate in the flavonoid/anthocyanin biosynthesis pathway. Among them, twelve DEGs were found to participate in the main pathway of flavonoid/anthocyanin biosynthesis (Table 1). Unigene0017118, Unigene0016797, Unigene0030889, and Unigene0012475 were annotated as chalcone synthase (*CHS*), chalcone isomerase (*CHI*), dihydroflavonol 4-reductase (*DFR*), and anthocyanidin synthase (*ANS*), respectively. All of them up-regulated in the red leaf stage more than in the green leaf stage. Unigene0014577 and Unigene0040486 were annotated as leucoanthocyanidin reductase (*LAR*) and anthocyanidin reductase (*ANR*), which showed a higher expression in the green leaf stage than in the red leaf stage. Both Unigene0041187 and Unigene0041186 were annotated as flavonoid 3′-hydroxylase (*F3′H*), which up-regulated in the green leaf stage, while Unigene0054556 and Unigene0052849 were both annotated as flavonol synthase (*FLS*), which showed high expression in the red leaf stage.

### 2.4. Identification of MYBs Involved in Anthocyanin Biosynthesis

**Figure 3:**
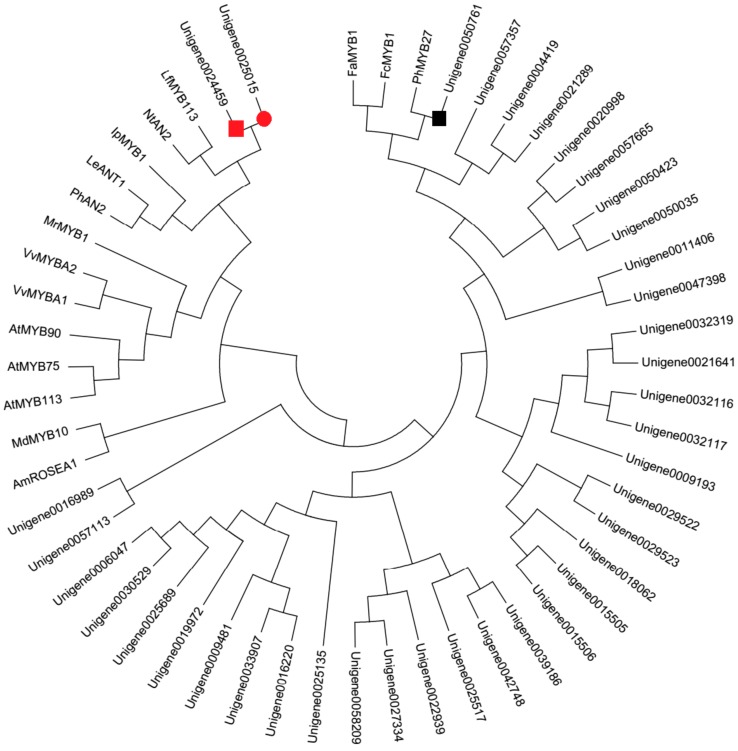
Phylogenetic relationships among putative MYBs in *P. qiui* and anthocyanin MYB regulators in other species. The phylogenetic tree was built using the neighbor-joining method using MEGA Version 6, and the bootstrap value was set to 1000. GenBank accession numbers were as follows: LeANT1 (AAQ55181.1), PhAN2 (BAP28593.1), IpMYB1 (ABW69685.1), LfMYB113 (ACO52472.1), NtAN2 (AQM49950.1), MrMYB1 (ADG21957.1), VvMYBA2 (ABL14065.1), VvMYBA1 (BAD18977.1), AtMYB75 (NP_176057.1), AtMYB90 (NM_105310), AtMYB113 (NP_176811.1), MdMYB10 (ACQ45201.1), AmROSEA1 (ABB83826.1), FaMYB1 (AF401220), FcMYB1 (ADK56163), and PhMYB27 (KF985023). The red square was the putative anthocyanin-activating MYB, and the black box was the putative anthocyanin repressor in *P. qiui*. The red circle was the putative anthocyanin-activating MYB in tree peony ‘Man Yuan Chun Guang’ [20].

Apart from enzyme genes, forty MYB genes were identified from DEGs in this study (Supplementary Table S3). In order to identify the candidate MYBs involved in anthocyanin biosynthesis, the phylogenetic analysis with the deduced amino acid sequences of these forty MYBs and other MYBs involved in anthocyanin biosynthesis was conducted (Figure 3). The phylogenetic tree showed that Unigene0024459 grouped into an anthocyanin promoting MYB clade, which included *Arabidopsis thaliana* AtMYB75 and AtMYB113, *Nicotiana tabacum* NtAN2, *Petunia hybrid* PhAN2, *Lycopersicon esculentum* LeANT1, *Vitis vinifera* VvMYBA1 and VvMYBA2, *Antirrhinum majus* AmROSEA1, *Myrica rubra* MrMYB1, *Malus domestica* MdMYB10, etc., while Unigene0050761 was in the same clade with FaMYB1, FcMYB1, and PhMYB27, which were proven to be the repressor of anthocyanin biosynthesis [16].

**Figure 4:**
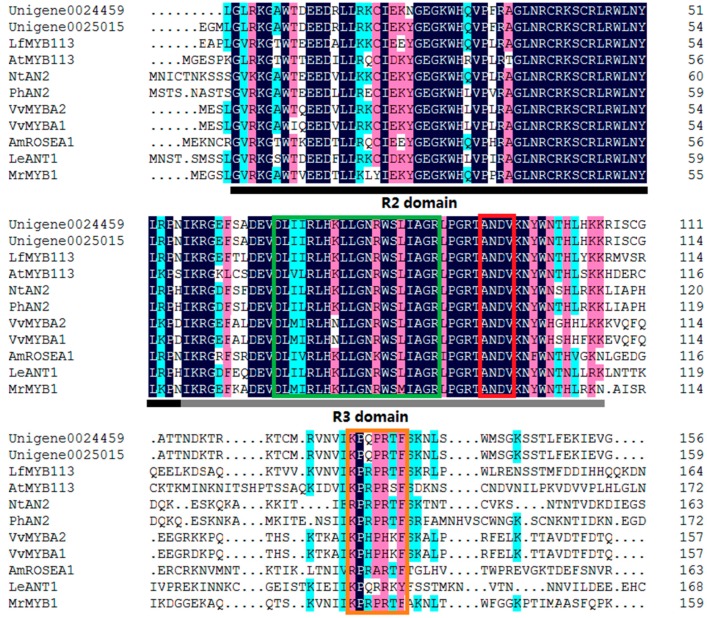
The deduced amino acid sequence alignment of Unigene0024459 with MYBs which specifically regulate anthocyanin biosynthesis in other species. Unigene0025015 is the MYB which was found in tree peony ‘Man Yuan Chun Guang’ [20]. Alignment was conducted using DNAMAN Version 6; R2 and R3 domains are indicated. The red box shows the ANDV motif, the orange box shows motif 6, and the green box shows the motifs that were involved in the bHLH interaction.

The alignment analysis of the deduced amino acid sequence of Unigene0024459 with those of other MYBs related to anthocyanin biosynthesis was conducted. Like other MYBs, Unigene0024459 contained the highly-conserved R2R3 domain at the N-terminus (Figure 4). All of them contained a conservative [K/R]Pxxx[K/T][F/Y] sequence in the C-terminal. This short, the conserved sequence was part of the signature motif for MYBs that positively regulated anthocyanin biosynthesis in vegetative or reproductive tissues [17]. Additionally, another conservative ANDV sequence at position 90–93 in the R3 domain was found in Unigene0024459. This conserved sequence was also the signature motif for the anthocyanin-promoting MYBs [17], which indicated that Unigene0024459 was a R2R3 MYB and likely promoted the anthocyanin biosynthesis in *P. qiui*. Meanwhile, Unigene0024459 contained the motif of [D/E]Lx_2_[R/K]x_3_Lx_6_Lx_3_R in the R3 domain that is responsible for interaction with the R-like bHLH protein [18], suggesting that Unigene0024459 possibly recruited some R-like bHLH proteins as cofactors during transcriptional regulation.

**Figure 5:**
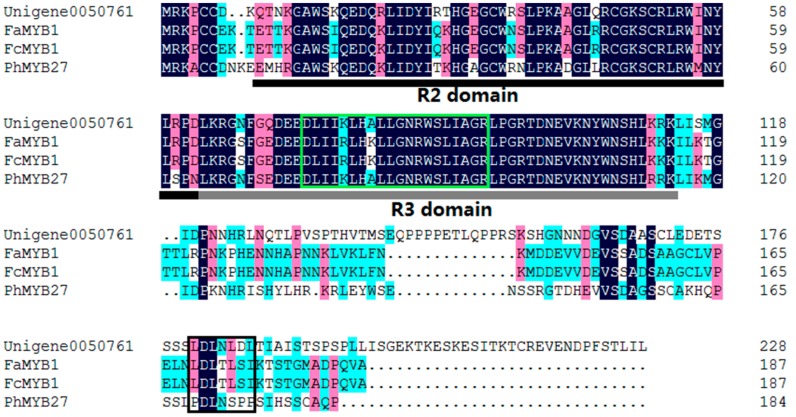
The deduced amino acid sequence alignment of Unigene0050761 with MYBs which repress anthocyanin biosynthesis in other species. Alignment was conducted using DNAMAN Version 6. R2 and R3 domains are indicated. The black box shows the EAR motif, and the green box shows the motifs that were involved in the bHLH interaction.

Alignment analysis of the deduced amino acid sequence showed that Unigene0050761 also contained the highly-conserved R2R3 domain at the N-terminus, and consensus sequence of LxLxL (Figure 5). This consensus sequence is the signature pattern of the EAR (for ethylene-responsive factor [ERF]-associated amphiphilic repression) motif, the predominant form of the transcriptional repressor motif identified in plants [19].

### 2.5. Identification bHLHs Involved in Anthocyanin Biosynthesis

**Figure 6:**
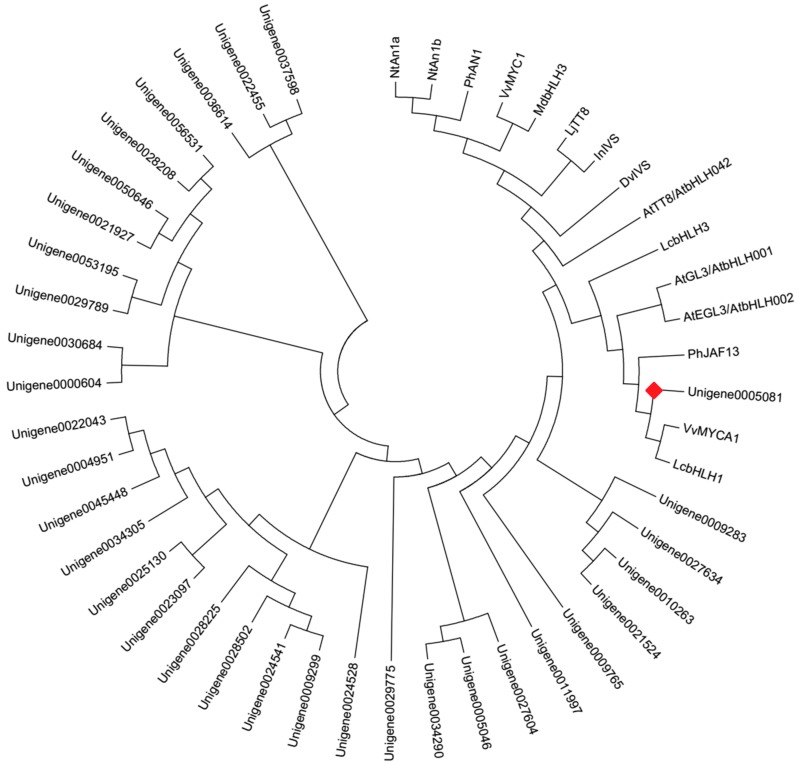
Phylogenetic relationships among putative bHLHs in *P. qiui* and anthocyanin bHLH regulators in other species. The phylogenetic tree was built using the neighbor-joining method using MEGA Version 6, and the bootstrap value was set to 1000. GenBank accession numbers were as follows: NtAn1a (AEE99257), NtAn1b (AEE99258), PhAN1 (AAG25927), VvMYC1 (ACC68685), MdbHLH3 (ADL36597), LjTT8 (BAH28881), InIVS (BAE94394), DvIVS (AB601005), AtTT8 (CAC14865), AtGL3 (NP 680372), AtEGL3 (NP 176552), PhJAF13 (AAC39455), VvMYCA1 (ABM92332), LcbHLH3 (APP94124.1), and LcbHLH1 (APP94122.1). The red box was the putative anthocyanin bHLH regulator in *P. qiui*.

**Figure 7:**
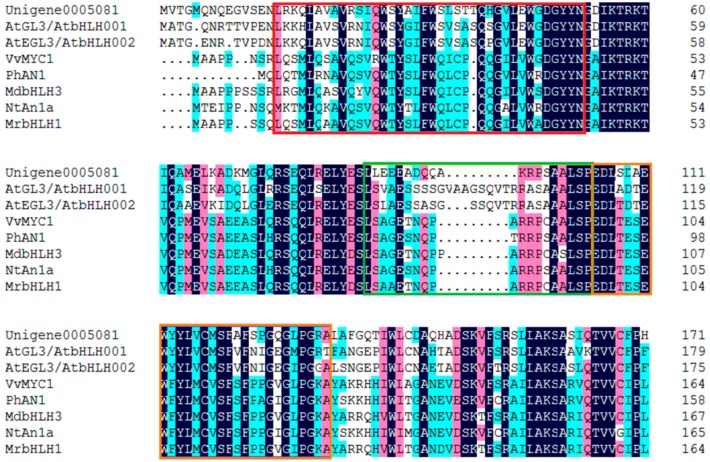
The deduced amino acid sequence alignment of Unigene0005081 with bHLHs which regulate anthocyanin biosynthesis in other species. Alignment was conducted using DNAMAN Version 6. The red box shows the box 11 motif, the green box shows the box 18 motif, and the orange box shows the box 13 motif.

Forty bHLH genes were identified from DEGs (Supplementary Table S4) in this study. The phylogenetic analysis with the deduced amino acid sequences showed that Unigene0005081 grouped into an anthocyanin biosynthesis-related bHLH clade, which included *Arabidopsis thaliana* AtGL3, AtEGL3, and AtTT8, *Nicotiana tabacum* NtAn1a and NtAn1b, *Petunia hybrida* PhAN1 and PhJAF13, *Vitis vinifera* VvMYCA1 and VvMYC1, *Malus domestica* MdbHLH3, *Dahlia pinnata* DvIVS, etc. (Figure 6). The deduced amino acid sequence alignment showed that Unigene0005081 contained box 11, 18 and 13 domain at the N-terminus (Figure 7), which was important for the interaction of bHLH and MYB [21]. These indicated that Unigene0005081 was likely involved in regulating the anthocyanin biosynthesis in *P. qiui*.

### 2.6. Identification WD40 Involved in Anthocyanin Biosynthesis

**Figure 8:**
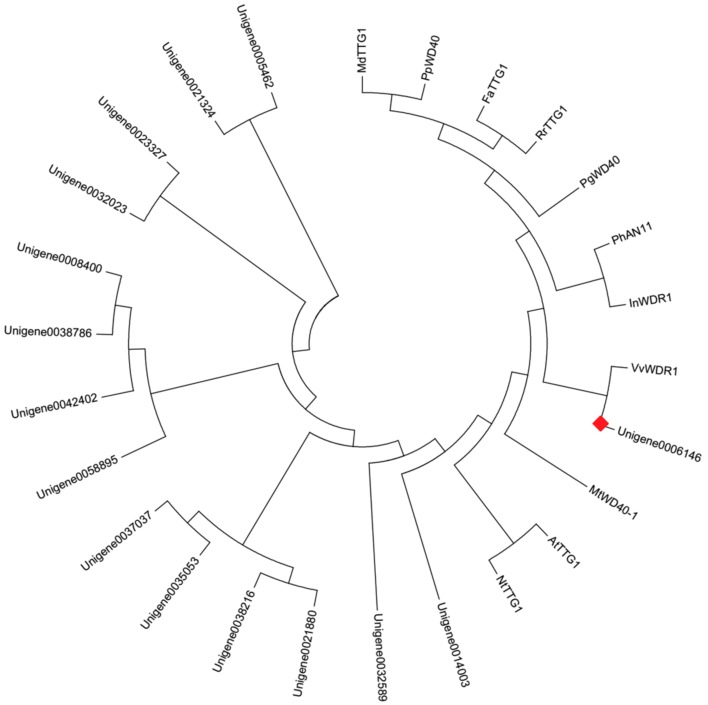
Phylogenetic relationships among putative WD40 in *P. qiui* and anthocyanin WD40 regulators in other species. The phylogenetic tree was built using the neighbor-joining method using MEGA Version 6, and the bootstrap value was set to 1000. GenBank accession numbers were as follows: MdTTG1 (ADI58759.1), PpWD40 (ADU24044.1), FaTTG1 (AFL02466.1), RrTTG1 (AFY23208.1), PgWD40 (ADV40946), PhAN11 (AAC18914), InWDR (AB232779.1), VvWDR1 (ABF66625), MtWD40-1 (ABW08112), AtTTG1 (NP 197840), and NtTTG1 (ACJ06978.1). The red box was the putative anthocyanin WD40 regulator in *P. qiui*.

Apart from MYBs and bHLHs, 15 WD40 transcription factors were also found from DEGs (Supplementary Table S5). The phylogenetic analysis with the deduced amino acid sequences showed that Unigene0006146 was in the same clade with VvWDR1, PhAN11, MdTTG1, PgWD40, InWDR1, NtTTG1, and AtTTG1 (Figure 8). These WD40 transcription factors participated in regulating the anthocyanin biosynthesis. It predicted that Unigene0006146 likely participated in regulating the anthocyanin biosynthesis in *P. qiui.*

### 2.7. Quantitative RT-PCR Analysis of the DEGs Involved in Anthocythanin Biosynthesis

**Figure 9:**
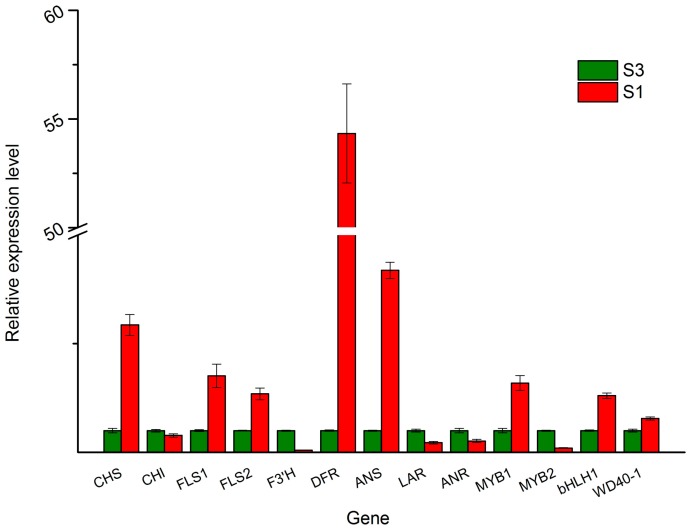
The expression patterns of DEGs involved in anthocyanin biosynthesis revealed by q-PCR. Unigene0017118 (*CHS*), Unigene0016797 (*CHI*), Unigene0030889 (*DFR*), Unigene0012475 (*ANS*), Unigene0014577 (*LAR*), Unigene0040486 (*ANR*) Unigene0041187 (*F3'H*), Unigene0054556 (*FLS1*), Unigene0052849 (*FLS2*), *MYB1* (Unigene0024459), *MYB2* (Unigene0050761), *bHLH1* (Unigene0005081), and *WD40-1* (Unigene0006146).

To test the reliability of the RNA sequencing data, 13 DEGs participated in tree peony leaf anthocyanin biosynthesis, including Unigene0017118 (*CHS*), Unigene0016797 (*CHI*), Unigene0030889 (*DFR*), Unigene0012475 (*ANS*), Unigene0014577 (*LAR*), Unigene0040486 (*ANR*) Unigene0041187 (*F3'H*), Unigene0054556 (*FLS1*), Unigene0052849 (*FLS2*), *MYB1* (Unigene0024459), *MYB2* (Unigene0050761), *bHLH1* (Unigene0005081), and *WD40-1* (Unigene0006146), which were selected for quantitative RT-PCR (q-PCR) analysis. Unigene0020903 (*ubiquitin*) was used as an internal control. The expression patterns revealed by q-PCR analysis were similar to those revealed by RNA sequencing for the same genes (Figure 9).

## 3. Discussion

The anthocyanin biosynthetic pathway is the best-characterized secondary metabolic pathway in plants, which is a specific branch of the flavonoid pathway [6]. CHS is responsible for the first committed step in the anthocyanin biosynthesis pathway. It condenses one molecule of 4-coumaroyl-CoA with three molecules of malonyl-CoA to produce the tetrahydroxy-chalcone, which is the precursor for the biosynthesis of flavonoids and anthocyanins. Dihydroflavonols (dihydrokaempferol and dihydroquercetin) are reduced to corresponding leucoanthocyanidins by the action of DFR, then ANS catalyzes the oxidation of the colorless leucoanthocyanidin to the corresponding colored anthocyanidin [22]. Additionally, leucoanthocyanidin can been converted to (+)-catechin catalyzed by LAR, and anthocyanidin can been converted to (−)-epicatechin by ANR [22]. Both (+)-catechin and (−)-epicatechin are colorless. The expression level of *CHS*, *DFR*, and *ANS* were higher in S1 (red leaf stage) than in S3 (non-red leaf stage) in the present study, which is similar in tree peony ‘Man Yuan Chun Guang’ [20] and ‘Jingrong’ [12], *P. delavayi* [14], and *P. rockii* [22], while the expression level of *LAR* and *ANR* were lower in S1. Owing to the high expression of *CHS*, *DFR*, *ANS,* and lower expression of *LAR* and *ANR* in the S1 stage, a large amount of anthocyanins were produced and accumulated in the S1 stage, and caused a purplish-red leaf (Figure 1). In the S3 stage, due to the low expression level of *CHS*, *DFR, ANS*, and the high expression level of *LAR* and *ANR*, few anthocyanins were produced and accumulated, which caused a very low anthocyanin level (Figure 1). These results are consistent with the hypothesis that the spring leaf red color fading of *P. qiui* may be caused by the expression level of *CHS*, *DFR*, *ANS*, *LAR*, and *ANR*, especially *CHS*, *DFR*, and *ANS*, which are mainly responsible for anthocyanin levels in tree peony (Figure 9). Additionally, through the sequence alignment, we found that the sequences of *CHS*, *DFR*, and *ANS* of the leaf have 99%, 98%, and 99% similarity with those of the flowers of tree peony, respectively [12,14,22]. This indicated that *CHS*, *DFR*, and *ANS* of the leaf might be the same as those of tree peony flowers.

As a branch of the flavonoid pathway, anthocyanin biosynthesis involves multiple structural genes [6]. The biosynthesis of anthocyanins is not only influenced by temperature, light, ultraviolet radiation, and other environmental factors, but also strictly regulated by transcription factors (TFs), including R2R3-MYB, bHLH, and WD40 [23]. Although these three types of TFs form a complex to coordinately regulate the transcription of the anthocyanin pathway structural genes, MYB TFs are widely reported to play a determinant role in anthocyanin biosynthesis in various dicotyledonous plants, such as *Antirrhinum majus* [24], cauliflower [25], petunia [26], asiatic-hybrid lily [27], monkey flowers [28], and *Phalaenopsis spp*. [29]. In this study, forty MYB genes were identified from DEGs in the RNA sequencing data. Through phylogenetic analysis with the deduced amino acid sequences of these forty MYBs and other MYBs involved in anthocyanin biosynthesis, Unigene0024459 (MYB1) grouped into an anthocyanin-activating MYB clade. Further alignment analysis shows that Unigene0024459 contained the highly-conserved R2R3 domain at the N-terminus, ANDV sequence in the R3 domain, and motif 6 in the C-terminal (Figure 4). The motif 6 was first defined by Stracke et al. as a KPRPR[S/T]F sequence in AtPAP1 and PhAN2, and then in CaA [30]. This motif is modified to [K/R]Pxxx[K/T][F/Y] in MdMYB1, GMYB10, AmROSAEA1, LeANT1, and VvMYBA1 [30]. So far, motif 6 is the only one found in the anthocyanin-activating MYB regulators in dicotyledonous plants [17]. Another convenient identifier for an anthocyanin-activating MYB appears to be ANDV (in over 90% of cases) with slight variations to become [A/S/G]N[D/V/N]V at position 90–93 in the R2R3 domain [17]. This motif was also found in Unigene0024459 (Figure 4). Additionally, Unigene0024459 contains the amino acid motif [D/E]Lx_2_[R/K]x_3_Lx_6_Lx_3_R, which is required for interaction with bHLH partners [18]. The q-PCR results showed that the expression level of Unigene0024459 was much higher in the red leaf stage than in the green leaf stage (Figure 9). Based this information, it indicated that Unigene0024459 (MYB1) might be an anthocyanin-activating R2R3-MYB, and possibly recruited some R-like bHLH proteins as cofactors during transcriptional regulation. In tree peony ‘Man Yuan Chun Guang’, one MYB (Unigene0025015) was also found by our team [20], which has a very close phylogenetic relationship, and almost the same sequences with Unigene0024459 (Figure 6 and Figure 7). Therefore, these two unigenes seem to be the same MYB, and this MYB was the putative anthocyanin-activating R2R3-MYB in tree peony.

In addition to anthocyanin-activating MYB regulators, several negative regulators of anthocyanin synthesis were also isolated, including single-domain R3-MYB and R2R3-MYB proteins [31,32,33]. The R2R3-MYB repressors contain domains in their C-terminus required for their repressive activity, such as the ERF motif (LxLxL or DLNxxP), which actively represses transcription and may involve the recruitment of chromatin remodeling factors [19]. R2R3-MYB proteins of this type that repress anthocyanin biosynthesis include Fa/Fc-MYB1 from strawberry [34,35] and PhMYB27 from petunia [26]. Unigene0050761 was in the same clade with FaMYB1, FcMYB1 and PhMYB27 (Figure 3) in this study. Alignment analysis of the deduced amino acid sequence shows that Unigene0050761 also contained the highly-conserved R2R3 domain at the N-terminus, and a LxLxL type of EAR motif in the amino-terminal region, which is also found in FaMYB1 and FcMYB1 [34,35]. Additionally, Unigene0050761 contains the amino acid motif [D/E]Lx2[R/K]x3Lx6Lx3R, which is required for interaction with bHLH partners [18]. The expression level of Unigene0050761 (MYB2) was much lower in the red leaf stage than in the green leaf stage in q-PCR (Figure 9). These implied that Unigene0050761 was likely an anthocyanin-repressor, and possibly recruited some R-like bHLH proteins as cofactors during transcriptional regulation.

Based on these information, we present a model of the leaf red color fading in tree peony: In spring, owing to some factors (such as temperature) induction, anthocyanin-repressor (MYB2) was activated gradually, incorporated into MBW activation complexes (MYB1 + bHLH1 + WD40-1) through the replacement of MYB1, and converted them into MBW-repressive complexes (MYB2 + bHLH1 + WD40-1) [33]. Then, the expression levels of *CHS*, *DFR* and *ANS* reduced by the regulation of MYB2 + bHLH1 + WD40-1 complexes (Figure 9), and caused anthocyanin levels to decrease from S1 to S3 in tree peony (Figure 1). Further study is needed to verify this model.

## 4. Materials and Methods

### 4.1. Plant Material

*P. qiui* plants were grown under field conditions in Northwest A and F University, Yangling Shaanxi, China. Leaf samples were collected in the morning during March and April 2017 (Figure 1). All samples were immediately frozen in liquid nitrogen, then stored at −80 °C for RNA extraction and anthocyanin analysis.

### 4.2. Anthocyanin Level Measurement

Freeze-dried leaves were finely ground and 0.5 g was extracted with 10 mL acidic methanol (0.1% hydrochloric acid) at 4 °C in darkness for 24 h, then suspended by ultrasonic agitation for 1 h. After centrifugation at 5000 rpm for 1 min, the supernatant was filtered using a 0.22 µm membrane filter (Shanghai Shupei Experimental Instrument Company, Shanghai, China). The absorbance was measured at 530 nm and 657 nm using a UV–VIS spectrophotometer (UV-3802, Unico, Suite E Dayton, OH, USA). Anthocyanin content was quantified using the equation A530−0.25 × A657. Three biological replicates were performed.

### 4.3. RNA Extraction, Library Construction, De Novo Assembly, and Gene Annotation

Total RNA was extracted from 1 mg pooled samples obtained by a homogenizer (TissueLyzer; Qiagen, Valencia, CA, USA) using the Quick RNA Isolation Kit (Bioteke Corporation, Beijing, China) according to the manufacturer’s protocols. RNase-free DNase I (Tiangen; Beijing, China) was applied to remove the contaminating genomic DNA. The RNA purity was determined with a NanoDrop 2000 spectrophotometer (NanoDrop Technologies; Wilmington, DE, USA), and 2% agarose gels were run to verify RNA integrity. The mRNA was enriched with oligo (dT)-rich magnetic beads, then the enriched mRNA was split into short sections using a fragmentation buffer and reverse-transcribed into cDNA with random primers. Second-strand cDNA was synthesized by DNA polymerase I, RNase H, dNTP, and buffer, then the cDNA fragments were purified with a QiaQuick PCR extraction kit, end repaired, poly(A) added, and ligated to Illumina sequencing adapters. The ligation products were size-selected by agarose gel electrophoresis, PCR amplified, and sequenced using Illumina HiSeqTM 4000 by Gene Denovo Biotechnology Co. (Guangzhou, China). Three biological replicates were used in this section.

The raw reads (which have been uploaded in the NCBI’s Sequence Read Archive, SRP123262) were first filtered by removing adaptor sequences, reads containing more than 10% of unknown nucleotides (N) and low-quality reads containing more than 50% of low quality (*Q*-value ≤ 10) bases. The remaining high-quality reads were assembled de novo using Trinity (https://github.com/trinityrnaseq/trinityrnaseq/wiki) [15]. During assembly, the reads from all three libraries were combined, and then ran in Trinity [15]. The longest transcript (from alternative splicing transcripts) was selected as the unigene in this study.

To annotate the unigenes, the resulting unigenes were aligned to the public databases, including the NCBI non-redundant protein (Nr) database (http://www.ncbi.nlm.nih.gov), the Swiss-Prot protein database (http://www.expasy.ch/sprot), the Kyoto Encyclopedia of Genes and Genomes (KEGG) database (http://www.genome.jp/kegg), and the COG/KOG database (http://www.ncbi.nlm.nih.gov/COG) using Blastx (*E*-value < 0.00001). Protein functional annotations could be obtained according to the best alignment results.

### 4.4. Identification and Analysis of DEGs

The unigene expression was calculated and normalized to RPKM (reads per kb per million reads) [36]. The false discovery rate (FDR) control method was used to identify the threshold of the *p*-value in multiple tests in order to compute the significance of the differences in transcript abundance. Here, only unigenes with an absolute value of log2 ratio > 1 and an FDR significance score < 0.05 were used to determine significant differences in gene expression. The confirmed DEGs were subjected to GO functional enrichment analysis and KEGG pathway analysis. Then, based on NR, NT, and Swiss-Prot annotations, GO functional analysis, and KEGG pathway analysis, the DEGs involved in leaf coloration were screened for up/down-regulated unigenes, and used for further analysis.

### 4.5. Phylogentic Tree and Sequence Alignment

The amino acid sequences of the MYB, bHLH, and WD40 transcription factors were acquired from the NCBI non-redundant protein (Nr) database (http://www.ncbi.nlm.nih.gov). Amino acid sequences were aligned and a phylogenetic tree image was generated using MEGA 6. The neighbor-joining method with 1000 bootstrap replicates was used. The sequences alignment was conducted by DNAMAN Version 6.

### 4.6. Quantitative RT-PCR (q-PCR) Analysis

Total RNA was extracted from leaves in the S1 and S3 stages, respectively. After treatment with RNase-free DNase I (Tiangen) according to the user’ manual, 1 μg of total RNA was reverse-transcribed to first-strand cDNA using the PrimeScript RT reagent kit (Takara; Otsu, Japan). Using SYBR Premix Ex Taq (Takara) on StepOnePlus Real-Time PCR system (Applied Biosystems, Waltham, MA, USA), q-PCR experiments were performed. Primers used are listed in Supplementary Table S1. The PCR program was carried out with an initial step of 95 °C for 30 s, and 40 cycles of 95 °C for 5 s, 55 °C for 30 s, 72 °C for 30 s; then 95 °C for 15 s, 60 °C for 1 min, and 95 °C for 15 s for the dissociation stage. The ubiquitin (Unigene0020903) was used to normalize the q-PCR data. The relative expression levels of genes were calculated by the 2^−∆∆*C*T^ comparative threshold cycle (*C*_t_) method. The statistical *p*-value was generated by the paired *t*-test. The statistical significance was defined as *p* < 0.05. Three biological replicates were performed for each gene.

## 5. Conclusions

The results indicate that Unigene0024459 is likely the R2R3-MYB that promotes anthocyanin biosynthesis; Unigene0050761 is likely the R2R3-MYB that represses anthocyanin biosynthesis; Unigene0005081 and Unigene0006146 are likely the bHLH and WD40 that participate in regulating anthocyanin biosynthesis, respectively.

## Figures and Tables

**Table 1:** Putative flavonoid/anthocyanin structural genes identified from differentially-expressed genes.

Gene ID	log2 Ratio (S3/S1)	FDR	Annotation
Unigene0055753	−10.26229064	1.81 × 10^−25^	Chalcone synthase (*Petunia x hybrida*)
Unigene0017118	−1.778044372	4.66 × 10^−40^	Chalcone synthase (*Paeonia suffruticosa*)
Unigene0053168	10.49914828	2.90 × 10^−11^	Chalcone isomerase (*Paeonia lactiflora*)
Unigene0016797	−1.255285418	5.88 × 10^−28^	Chalcone isomerase (*Paeonia suffruticosa*)
Unigene0052849	−2.566247123	4.90 × 10^−3^	Flavonol synthase (*Paeonia lactiflora*)
Unigene0054556	−2.6979596	8.02 × 10^−4^	Flavonol synthase (*Paeonia lactiflora*)
Unigene0041186	1.051737457	4.40 × 10^−2^	Flavanone 3′-hydroxylase (*Paeonia suffruticosa*)
Unigene0041187	1.616420273	2.53 × 10^−35^	Flavanone 3′-hydroxylase (*Paeonia suffruticosa*)
Unigene0030889	−4.77004356	9.17 × 10^−206^	Dihydroflavonol-4-reductase (*Paeonia suffruticosa*)
Unigene0012475	−3.959416654	2.24 × 10^−147^	Anthocyanidin synthase (*Paeonia suffruticosa*)
Unigene0014577	1.167626811	1.70 × 10^−4^	Leucoanthocyanidin reductase (*Cephalotus follicularis*)
Unigene0040486	9.329871068	5.28 × 10^−5^	Anthocyanidin reductase (*Malus domestica*)
Unigene0049627	−4.379465382	2.57 × 10^−6^	Caffeoyl-CoA *O*-methyltransferase
Unigene0010574	−2.325935914	4.03 × 10^−27^	Caffeoyl-CoA *O*-methyltransferase
Unigene0034757	2.750132254	2.90 × 10^−62^	Caffeoyl-CoA *O*-methyltransferase
Unigene0034758	1.774744649	1.62 × 10^−4^	Caffeoyl-CoA *O*-methyltransferase
Unigene0057244	−2.050989001	8.69 × 10^−43^	Caffeoyl-CoA *O*-methyltransferase
Unigene0034759	2.495157744	3.70 × 10^−76^	Caffeoyl-CoA *O*-methyltransferase
Unigene0031564	−2.848573896	4.97 × 10^−6^	Coumaroylquinate 3-monooxygenase
Unigene0054125	5.051629256	1.30 × 10^−8^	Coumaroylquinate 4′-monooxygenase
Unigene0035320	2.392910853	3.76 × 10^−69^	Coumaroylquinate 5′-monooxygenase
Unigene0045080	3.096711889	3.19 × 10^−59^	Shikimate *O*-hydroxycinnamoyltransferase
Unigene0058586	9.136649403	6.65 × 10^−3^	Shikimate *O*-hydroxycinnamoyltransferase
Unigene0029038	2.210473634	7.78 × 10^−67^	Shikimate *O*-hydroxycinnamoyltransferase
Unigene0052531	−1.54109082	1.15 × 10^−13^	Shikimate *O*-hydroxycinnamoyltransferase

## Supplementary Materials

Supplementary Materials are available online; Figure S1 and Tables S1–S5.
