# Supplementary material for: Transcriptomic Analysis Reveals Transcription Factors Related to Leaf Anthocyanin Biosynthesis in Paeonia qiui
Source: Molecules. 2017 Dec 8;22(12):2186. doi: 10.3390/molecules22122186 (PMC6149671; doi:10.3390/molecules22122186)
Supplement: Supplementary file 1 [file molecules-22-02186-s001.pdf]

Transcriptomic analysis reveals transcription factors related to leaf anthocyanin biosynthesis in *Paeonia qiui*

Jianrang Luo[1,2], Jingjing Duan[1,2], Dan Huo[1,2], Qianqian Shi[1,2], Lixin Niu[1,2,*] and Yanlong Zhang[1,2,*]

[1] College of Landscape Architecture and Art, Northwest A&F University, Yangling 712100, Shaanxi, China
[2] National Engineering Research Center for Oil Peony, Yangling 712100, Shaanxi, China
* Correspondence: niulixin@nwafu.edu.cn; zhangyanlong@nwafu.edu.cn; Tel: +86-029-87082878

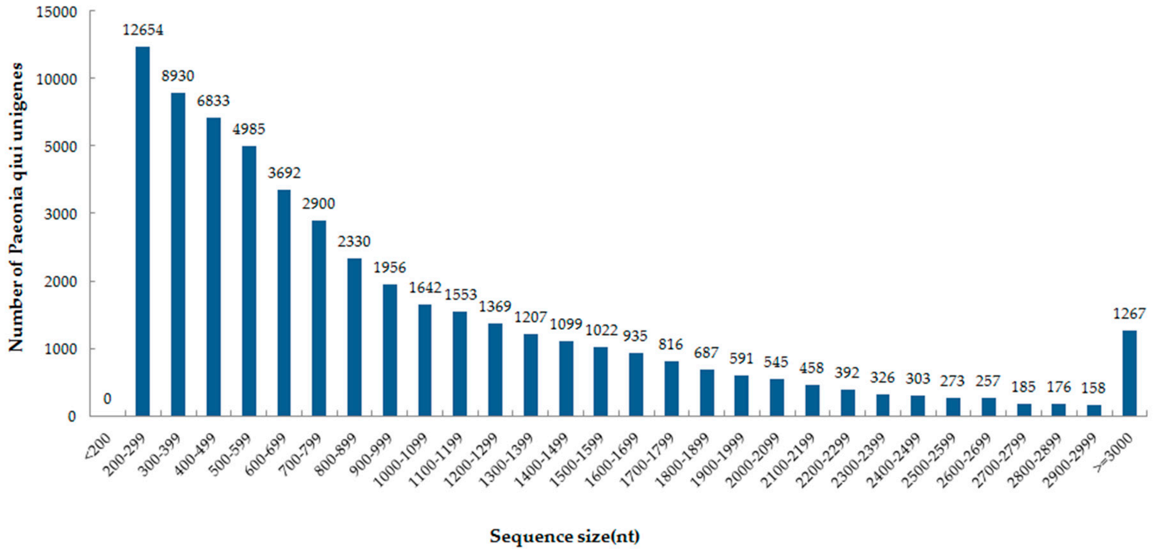

Figure S1: Size distribution of unigenes

Table S1. Primers used for q-PCR analysis

**Gene ID**	**Forward primer（5′—3′）**	**Reverse primer （5′—3′）**
Unigene0006146	TCCTCACTTCGCAAATCCTCC	TCAACCACACCCCTCTCCACA
Unigene0005081	GCTTCAATCCTTACCGATACA	TCAATCAAAACATCCCGCTCT
Unigene0024459	GCTGTAGATTGAGGTGGTTGA	TCTTCTTGTGTAGGTGTGTGT
Unigene0050761	TTGACCCTAATAACCATCGCC	TCAAGATTCAAGTCAAGCGAG
Unigene0054556	CATAATCTCGCTATCTCATCC	GCATAATTCTCCTTCTCCTCC
Unigene0052849	TCCACAACCTGAATTGGCACT	CAGCCCAGTTACCTTCTTTCC
Unigene0017118	CTGCTATCATCATTGGTTCGG	GTCGCTTGTAGTTTTTCGGGT
Unigene0016797	CTATTCTTTTCACACAGACACC	TGCTTCCCTATGATCGACTCC
Unigene0030889	TTCATCGGTTCATGGCTTGTC	CTCTCCGCACAGTTTTAGCC
Unigene0012475	ACCAGCATCACCAACATCTTC	GCATATTTCTCCTTCTCTTCC
Unigene0014577	TTGCTTCTTGGCCCTACTTTG	GTCCTCGGTTATGGTTTGTCT
Unigene0041187	GATGTTGATAGCGAGGGTGG	AGGAAGGGGAGATTTGGGAG
Unigene0040486	ATGGCTGGTCCTTCACTTACT	CGAAATGGATATGGAACCTGA

Table S2. KEGG pathway enrichment of differentially expressed genes

No.	Pathway	DEGs genes with pathway annotation (2333)		All genes with pathway annotation (6335)		*p*-value	Pathway ID
1	Ribosome	248	(10.63%)	521	(8.22%)	9.81×10^{-8}	ko03010
2	Phenylpropanoid biosynthesis	81	(3.47%)	143	(2.26%)	8.74×10^{-7}	ko00940
3	Plant hormone signal transduction	113	(4.84%)	227	(3.58%)	3.36×10^{-5}	ko04075
4	Starch and sucrose metabolism	129	(5.53%)	266	(4.20%)	4.64×10^{-5}	ko00500
5	Linoleic acid metabolism	19	(0.81%)	25	(0.39%)	7.50×10^{-5}	ko00591
6	DNA replication	48	(2.06%)	84	(1.33%)	0.000109739	ko03030
7	Nitrogen metabolism	29	(1.24%)	45	(0.71%)	0.00014794	ko00910
8	Pentose and glucuronate interconversions	57	(2.44%)	108	(1.70%)	0.000472719	ko00040
9	Stilbenoid, diarylheptanoid and gingerol biosynthesis	17	(0.73%)	24	(0.38%)	0.000731129	ko00945
10	Terpenoid backbone biosynthesis	41	(1.76%)	74	(1.17%)	0.000814305	ko00900
11	Glycine, serine and threonine metabolism	48	(2.06%)	90	(1.42%)	0.000955863	ko00260
12	Plant-pathogen interaction	110	(4.71%)	238	(3.76%)	0.001548023	ko04626
13	Fatty acid elongation	25	(1.07%)	42	(0.66%)	0.002246963	ko00062
14	Cyanoamino acid metabolism	39	(1.67%)	73	(1.15%)	0.002667798	ko00460
15	Galactose metabolism	42	(1.80%)	80	(1.26%)	0.002878465	ko00052
16	Monoterpenoid biosynthesis	9	(0.39%)	11	(0.17%)	0.003047899	ko00902
17	Flavonoid biosynthesis	25	(1.07%)	43	(0.68%)	0.003510991	ko00941
18	Sesquiterpenoid and triterpenoid biosynthesis	15	(0.64%)	23	(0.36%)	0.005251659	ko00909
19	alpha-Linolenic acid metabolism	32	(1.37%)	60	(0.95%)	0.00643939	ko00592
20	Glutathione metabolism	51	(2.19%)	110	(1.74%)	0.02429231	ko00480
21	Biotin metabolism	13	(0.56%)	22	(0.35%)	0.02770014	ko00780
22	Carotenoid biosynthesis	21	(0.90%)	40	(0.63%)	0.03062768	ko00906
23	Fatty acid metabolism	45	(1.93%)	99	(1.56%)	0.04693405	ko01212
24	Flavone and flavonol biosynthesis	3	(0.13%)	3	(0.05%)	0.04990584	ko00944

No.	Pathway	DEGs genes with pathway annotation (2333)		All genes with pathway annotation (6335)		p-value	Pathway ID
25	Synthesis and degradation of ketone bodies	6	(0.26%)	9	(0.14%)	0.06793747	ko00072
26	Phenylalanine metabolism	24	(1.03%)	50	(0.79%)	0.06874378	ko00360
27	Zeatin biosynthesis	10	(0.43%)	18	(0.28%)	0.08202637	ko00908
28	Alanine, aspartate and glutamate metabolism	30	(1.29%)	66	(1.04%)	0.09248293	ko00250
29	Tropane, piperidine and pyridine alkaloid biosynthesis	17	(0.73%)	35	(0.55%)	0.1034937	ko00960
30	Taurine and hypotaurine metabolism	7	(0.30%)	12	(0.19%)	0.1079198	ko00430
31	Fatty acid biosynthesis	23	(0.99%)	50	(0.79%)	0.1153628	ko00061
32	Diterpenoid biosynthesis	14	(0.60%)	29	(0.46%)	0.1387011	ko00904
33	Isoquinoline alkaloid biosynthesis	14	(0.60%)	29	(0.46%)	0.1387011	ko00950
34	Degradation of aromatic compounds	8	(0.34%)	15	(0.24%)	0.1451353	ko01220
35	Tyrosine metabolism	26	(1.11%)	59	(0.93%)	0.1532415	ko00350
36	Phenylalanine, tyrosine and tryptophan biosynthesis	26	(1.11%)	59	(0.93%)	0.1532415	ko00400
37	Inositol phosphate metabolism	33	(1.41%)	77	(1.22%)	0.1622349	ko00562
38	Glyoxylate and dicarboxylate metabolism	51	(2.19%)	123	(1.94%)	0.1629265	ko00630
39	Pentose phosphate pathway	36	(1.54%)	85	(1.34%)	0.1707624	ko00030
40	Phosphatidylinositol signaling system	32	(1.37%)	75	(1.18%)	0.1747285	ko04070
41	Base excision repair	31	(1.33%)	73	(1.15%)	0.188132	ko03410
42	Cysteine and methionine metabolism	57	(2.44%)	140	(2.21%)	0.1901492	ko00270
43	Homologous recombination	35	(1.50%)	84	(1.33%)	0.2075322	ko03440
44	Limonene and pinene degradation	4	(0.17%)	7	(0.11%)	0.2309155	ko00903
45	Mismatch repair	29	(1.24%)	70	(1.10%)	0.2471647	ko03430
46	Endocytosis	98	(4.20%)	251	(3.96%)	0.2485607	ko04144
47	Biosynthesis of unsaturated fatty acids	21	(0.90%)	50	(0.79%)	0.2671184	ko01040
48	Fatty acid degradation	22	(0.94%)	53	(0.84%)	0.2827942	ko00071
49	Tryptophan metabolism	12	(0.51%)	28	(0.44%)	0.3159318	ko00380
50	Ascorbate and aldarate metabolism	22	(0.94%)	54	(0.85%)	0.3204958	ko00053

No.	Pathway	DEGs genes with pathway annotation (2333)		All genes with pathway annotation (6335)		p-value	Pathway ID
51	Ether lipid metabolism	18	(0.77%)	44	(0.69%)	0.3382354	ko00565
52	Carbon fixation in photosynthetic organisms	56	(2.40%)	145	(2.29%)	0.3549311	ko00710
53	Brassinosteroid biosynthesis	7	(0.30%)	16	(0.25%)	0.3689303	ko00905
54	Steroid biosynthesis	15	(0.64%)	37	(0.58%)	0.3776071	ko00100
55	Circadian rhythm - plant	17	(0.73%)	43	(0.68%)	0.4115331	ko04712
56	Monobactam biosynthesis	4	(0.17%)	9	(0.14%)	0.437184	ko00261
57	Photosynthesis	42	(1.80%)	112	(1.77%)	0.4766864	ko00195
58	Carbon metabolism	153	(6.56%)	414	(6.54%)	0.4968967	ko01200
59	Pyrimidine metabolism	67	(2.87%)	181	(2.86%)	0.5072089	ko00240
60	Thiamine metabolism	8	(0.34%)	21	(0.33%)	0.5340385	ko00730
61	Butanoate metabolism	8	(0.34%)	21	(0.33%)	0.5340385	ko00650
62	Nucleotide excision repair	35	(1.50%)	95	(1.50%)	0.5378195	ko03420
63	Glycolysis / Gluconeogenesis	59	(2.53%)	161	(2.54%)	0.549408	ko00010
64	Nicotinate and nicotinamide metabolism	9	(0.39%)	24	(0.38%)	0.5495391	ko00760
65	Glycosphingolipid biosynthesis - globo series	5	(0.21%)	13	(0.21%)	0.5552117	ko00603
66	Arachidonic acid metabolism	6	(0.26%)	16	(0.25%)	0.5714135	ko00590
67	Cutin, suberine and wax biosynthesis	11	(0.47%)	30	(0.47%)	0.5758488	ko00073
68	Peroxisome	41	(1.76%)	113	(1.78%)	0.5836469	ko04146
69	Sulfur metabolism	13	(0.56%)	36	(0.57%)	0.5978298	ko00920
70	Other types of O-glycan biosynthesis	3	(0.13%)	8	(0.13%)	0.6150762	ko00514
71	Anthocyanin biosynthesis	3	(0.13%)	8	(0.13%)	0.6150762	ko00942
72	Amino sugar and nucleotide sugar metabolism	56	(2.40%)	157	(2.48%)	0.6488985	ko00520
73	Non-homologous end-joining	5	(0.21%)	15	(0.24%)	0.7016185	ko03450
74	Isoflavonoid biosynthesis	3	(0.13%)	9	(0.14%)	0.7040224	ko00943
75	Lipoic acid metabolism	2	(0.09%)	6	(0.09%)	0.7142406	ko00785
76	Arginine biosynthesis	19	(0.81%)	56	(0.88%)	0.7198241	ko00220

No.	Pathway	DEGs genes with pathway annotation (2333)		All genes with pathway annotation (6335)		p-value	Pathway ID
77	Glycerophospholipid metabolism	43	(1.84%)	124	(1.96%)	0.7223482	ko00564
78	Protein export	20	(0.86%)	60	(0.95%)	0.7554342	ko03060
79	Photosynthesis - antenna proteins	10	(0.43%)	31	(0.49%)	0.7599607	ko00196
80	Vitamin B6 metabolism	5	(0.21%)	16	(0.25%)	0.7608027	ko00750
81	Fructose and mannose metabolism	31	(1.33%)	92	(1.45%)	0.7677863	ko00051
82	Glycosaminoglycan degradation	7	(0.30%)	23	(0.36%)	0.8014944	ko00531
83	Propanoate metabolism	17	(0.73%)	53	(0.84%)	0.8050195	ko00640
84	Histidine metabolism	5	(0.21%)	17	(0.27%)	0.8106426	ko00340
85	Lysine degradation	13	(0.56%)	42	(0.66%)	0.8292116	ko00310
86	Sphingolipid metabolism	12	(0.51%)	39	(0.62%)	0.8294074	ko00600
87	Spliceosome	105	(4.50%)	306	(4.83%)	0.8401597	ko03040
88	Selenocompound metabolism	6	(0.26%)	21	(0.33%)	0.8444504	ko00450
89	Pyruvate metabolism	37	(1.59%)	114	(1.80%)	0.8590132	ko00620
90	Glycerolipid metabolism	24	(1.03%)	77	(1.22%)	0.8767001	ko00561
91	Lysine biosynthesis	5	(0.21%)	19	(0.30%)	0.8852201	ko00300
92	One carbon pool by folate	8	(0.34%)	29	(0.46%)	0.8922036	ko00670
93	Riboflavin metabolism	4	(0.17%)	16	(0.25%)	0.8960274	ko00740
94	Valine, leucine and isoleucine degradation	20	(0.86%)	67	(1.06%)	0.9078143	ko00280
95	Arginine and proline metabolism	18	(0.77%)	61	(0.96%)	0.9090556	ko00330
96	SNARE interactions in vesicular transport	17	(0.73%)	58	(0.92%)	0.9099296	ko04130
97	Porphyrin and chlorophyll metabolism	16	(0.69%)	55	(0.87%)	0.9110034	ko00860
98	mRNA surveillance pathway	57	(2.44%)	178	(2.81%)	0.924301	ko03015
99	Other glycan degradation	9	(0.39%)	34	(0.54%)	0.9272641	ko00511
100	Biosynthesis of amino acids	105	(4.50%)	317	(5.00%)	0.9290205	ko01230
101	Ribosome biogenesis in eukaryotes	50	(2.14%)	163	(2.57%)	0.959786	ko03008
102	Purine metabolism	70	(3.00%)	222	(3.50%)	0.9598967	ko00230

No.	Pathway	DEGs genes with pathway annotation (2333)		All genes with pathway annotation (6335)		p-value	Pathway ID
103	Glycosphingolipid biosynthesis - ganglio series	1	(0.04%)	8	(0.13%)	0.9746998	ko00604
104	Regulation of autophagy	12	(0.51%)	49	(0.77%)	0.9770373	ko04140
105	Folate biosynthesis	4	(0.17%)	21	(0.33%)	0.9776331	ko00790
106	Pantothenate and CoA biosynthesis	7	(0.30%)	32	(0.51%)	0.9777182	ko00770
107	Protein processing in endoplasmic reticulum	107	(4.59%)	336	(5.30%)	0.9782724	ko04141
108	beta-Alanine metabolism	12	(0.51%)	50	(0.79%)	0.9817576	ko00410
109	Proteasome	18	(0.77%)	71	(1.12%)	0.9857748	ko03050
110	Valine, leucine and isoleucine biosynthesis	4	(0.17%)	23	(0.36%)	0.9884576	ko00290
111	C5-Branched dibasic acid metabolism	1	(0.04%)	10	(0.16%)	0.989919	ko00660
112	Sulfur relay system	2	(0.09%)	15	(0.24%)	0.9901401	ko04122
113	ABC transporters	25	(1.07%)	97	(1.53%)	0.9925878	ko02010
114	Phagosome	27	(1.16%)	104	(1.64%)	0.993332	ko04145
115	RNA degradation	59	(2.53%)	204	(3.22%)	0.9936218	ko03018
116	2-Oxocarboxylic acid metabolism	17	(0.73%)	72	(1.14%)	0.9943988	ko01210
117	Glycosylphosphatidylinositol(GPI)-anchor biosynthesis	8	(0.34%)	41	(0.65%)	0.9951002	ko00563
118	Ubiquitin mediated proteolysis	60	(2.57%)	211	(3.33%)	0.9963919	ko04120
119	RNA polymerase	15	(0.64%)	73	(1.15%)	0.9991898	ko03020
120	N-Glycan biosynthesis	8	(0.34%)	49	(0.77%)	0.9995688	ko00510
121	Ubiquinone and other terpenoid-quinone biosynthesis	13	(0.56%)	70	(1.10%)	0.9997493	ko00130
122	Oxidative phosphorylation	50	(2.14%)	197	(3.11%)	0.9998066	ko00190
123	Basal transcription factors	13	(0.56%)	74	(1.17%)	0.9999212	ko03022
124	Citrate cycle (TCA cycle)	13	(0.56%)	75	(1.18%)	0.9999413	ko00020
125	RNA transport	74	(3.17%)	291	(4.59%)	0.9999914	ko03013
126	Aminoacyl-tRNA biosynthesis	8	(0.34%)	72	(1.14%)	0.9999998	ko00970

Table S3. MYBs identified from differentially expressed genes

GeneID	log2 Ratio(S3/S1)*	Annotation
Unigene0029523	-12.57036964	Myb domain protein 106 (*Theobroma cacao*)
Unigene0057113	-11.49290468	transcription factor MYB108 (*Vitis vinifera*)
Unigene0029522	-9.970046156	Myb domain protein 106 (*Theobroma cacao*)
Unigene0016989	-8.829383836	transcription factor MYB108-like (*Populus euphratica*)
Unigene0032117	-7.900304871	myb-related protein Myb4-like (*Populus euphratica*)
Unigene0032319	-6.93164243	myb-related protein Myb4-like (*Juglans regia*)
Unigene0011406	-6.137168382	transcription repressor MYB6 isoform X1 (*Theobroma cacao*)
Unigene0025135	-6.111096685	transcription factor MYB48 (*Vitis vinifera*)
Unigene0016220	-5.263146719	transcription factor MYB44-like (*Ziziphus jujuba*)
Unigene0032116	-4.832477733	myb-related protein Myb4-like (*Populus euphratica*)
Unigene0030529	-3.676597687	myb-related protein 3R-1 isoform X1 (*Vitis vinifera*)
Unigene0047398	-3.396310077	transcription factor MYB3-like (*Jatropha curcas*)
Unigene0024459	-3.356215515	MYB transcription factor 113 (*Liquidambar formosana*)
Unigene0057357	-3.23926588	myb-related protein 308-like (*Vitis vinifera*)
Unigene0006047	-3.163869449	myb-related protein 3R-1 isoform X1 (*Vitis vinifera*)
Unigene0004419	-2.655599465	transcription repressor MYB6-like (*Juglans regia*)
Unigene0021641	-2.603584268	myb-related protein Myb4-like (*Ziziphus jujuba*)
Unigene0020583	-2.448745375	R2R3 Myb protein, partial (*Zea mays*)
Unigene0018062	-2.350682055	MYB1 protein (*Paeonia suffruticosa*)
Unigene0019972	-2.028269696	myb-related protein 3R-1-like (*Ziziphus jujuba*)
Unigene0039186	-1.857530006	L10-interacting MYB domain-containing (*Nicotiana attenuata*)
Unigene0025689	-1.517664466	myb-related protein B-like (*Juglans regia*)
Unigene0022939	-1.509975137	transcription factor MYB86-like (*Gossypium hirsutum*)
Unigene0033907	-1.005823383	myb-like protein B (*Vitis vinifera*)
Unigene0042748	-1.003142265	L10-interacting MYB domain-containing (*Malus domestica*)
Unigene0020998	-1.000574525	R2R3-MYB transcription factor MYB2 (*Epimedium sagittatum*)
Unigene0025517	1.022010118	L10-interacting MYB domain-containing (*Vitis vinifera*)
Unigene0021289	1.112487772	myb-related protein 308-like (*Populus euphratica*)
Unigene0050761	1.144656074	transcription repressor MYB6 (*Ricinus communis*)
Unigene0025266	1.242155836	MYB transcription factor R2R3-like (*Populus tremuloides*)
Unigene0027334	2.45635767	MYB61 protein (*Betula platyphylla*)
Unigene0050423	2.477964054	transcription factor MYB46-like (*Populus euphratica*)
Unigene0015505	2.615240635	Myb DNA-binding domain-containing (*Cephalotusfollicularis*)
Unigene0050035	3.121526809	myb-related protein Hv33 (*Ipomoea nil*)
Unigene0009193	3.582518158	MYB-like 102 (*Theobroma cacao*)
Unigene0015506	3.63032412	myb-related protein 306-like (*Gossypium raimondii*)
Unigene0058209	3.7291144	transcription factor MYB82 (*Vitis vinifera*)
Unigene0057664	5.385523072	Myb_DNA-binding domain-containing (*Cephalotus follicularis*)
Unigene0057665	10.45703881	Myb_DNA-binding domain-containing (*Cephalotus follicularis*)
Unigene0009481	10.88310146	MYB transcriptional factor (*Populus tremula* × *P. tremuloides*)

* Data were the mean value of three biological replicates.

Table S4. bHLHs identified from differentially expressed genes

GeneID	log2 Ratio(S3/S1)	Annotation
Unigene0000604	-1.542977885	transcription factor bHLH140 (*Citrus sinensis*)
Unigene0000838	-1.184189305	transcription factor bHLH69 isoform X2 (*Vitis vinifera*)
Unigene0004951	-2.253690822	transcription factor bHLH68-like (*Juglans regia*)
Unigene0005046	-5.824634604	transcription factor bHLH94-like (*Juglans regia*)
Unigene0009283	-1.356731517	bHLH transcription factor (*Paeonia suffruticosa*)
Unigene0009299	3.01694928	transcription factor bHLH63 (*Vitis vinifera*)
Unigene0009765	-11.03103489	transcription factor bHLH93-like (*Juglans regia*)
Unigene0010263	1.190472009	transcription factor bHLH14 (*Theobroma cacao*)
Unigene0011997	-1.239357047	transcription factor bHLH104 isoform X1 (*Citrus sinensis*)
Unigene0021524	-10.39934147	transcription factor MYC4 (*Vitis vinifera*)
Unigene0021927	2.310369042	transcription factor bHLH36 (*Theobroma cacao*)
Unigene0022043	-2.507522348	transcription factor bHLH68 (*Ricinus communis*)
Unigene0022455	-4.607713173	transcription factor bHLH35-like (*Pyrus* × *bretschneideri*)
Unigene0023097	-3.228124521	transcription factor bHLH87 (*Theobroma cacao*)
Unigene0023465	-1.040981906	transcription factor bHLH113 isoform X1 (*Prunus mume*)
Unigene0024528	-1.817136179	basic helix-loop-helix 144-like protein transcript variant 1 (*Morus alba*)
Unigene0024541	-1.430788575	transcription factor bHLH74 isoform X2 (*Prunus mume*)
Unigene0025130	2.04495838	transcription factor bHLH117 (*Vitis vinifera*)
Unigene0027604	-9.020557667	transcription factor bHLH92 (*Juglans regia*)
Unigene0028208	-1.588286752	transcription factor bHLH51 (*Vitis vinifera*)
Unigene0028225	-1.066042545	transcription factor bHLH147 isoform X1 (*Vitis vinifera*)
Unigene0028502	-1.456043273	transcription factor bHLH79-like isoform X2 (*Nelumbo nucifera*)
Unigene0029775	-5.265879952	HLH domain-containing protein (*Cephalotus follicularis*)
Unigene0029789	-4.584853904	Serine/threonine-protein kinase WNK-related (*Theobroma cacao*)
Unigene0030684	-1.549969453	transcription factor bHLH128-like isoform X1 (*Juglans regia*)
Unigene0031383	2.345274781	BHLH transcription factor (*Medicago truncatula*)
Unigene0034290	-5.735383852	transcription factor bHLH70 isoform X2 (*Vitis vinifera*)
Unigene0034305	-2.982852155	transcription factor bHLH113 isoform X3 (*Vitis vinifera*)
Unigene0036614	-1.576885394	transcription factor bHLH140 (*Prunus mume*)
Unigene0036639	-1.163393004	transcription factor bHLH91 (*Ricinus communis*)
Unigene0037066	4.11563007	transcription factor bHLH18-like (*Solanum tuberosum*)
Unigene0037598	-7.711518195	transcription factor bHLH36 (*Ziziphus jujuba*)
Unigene0037850	-1.050886838	immunoglobulin A1 protease autotransporter isoform X1 (*Arachis ipaensis*)
Unigene0037852	-1.818022021	transcription factor bHLH25-like (*Vitis vinifera*)
Unigene0045448	-2.464557332	transcription factor bHLH110 (*Theobroma cacao*)
Unigene0050646	-7.156893165	transcription factor bHLH36 (*Theobroma cacao*)
Unigene0053195	-3.402985048	Serine/threonine-protein kinase WNK-related isoform 1 (*Theobroma cacao*)
Unigene0056531	-2.396829315	transcription factor bHLH106-like isoform X2 (*Juglans regia*)
Unigene0005081	-1.373426581	myc anthocyanin regulatory protein isoform X1 (*Vitis vinifera*)
Unigene0027634	-2.372379263	transcription factor MYC2 (*Vitis vinifera*)

* Data were the mean value of three biological replicates.

Table S5. WD40 identified from differentially expressed genes

GeneID	log2 Ratio(S3/S1)*	Annotation
Unigene0021880	-3.526584548	WD repeat-containing protein 76 isoform X2 (*Jatropha curcas*)
Unigene0038216	-3.493187425	WD repeat-containing protein 76 isoform X2 (*Jatropha curcas*)
Unigene0021324	-1.948580414	WD repeat-containing protein 43 isoform X2 (*Vitis vinifera*)
Unigene0042402	-1.626752223	WD repeat-containing protein 89 homolog (*Vitis vinifera*)
Unigene0014003	-1.611681795	mitogen-activated protein kinase-binding protein 1 isoform X3 (*Vitis vinifera*)
Unigene0035053	-1.551363637	WD repeat-containing protein 44 isoform X1 (*Cucumis melo*)
Unigene0032589	-1.532298896	WD40 domain-containing protein (*Cephalotus follicularis*)
Unigene0005462	-1.430489052	WD repeat-containing protein 43 isoform X1 (*Vitis vinifera*)
Unigene0006146	-1.347867295	WD40 protein (*Paeonia suffruticosa*)
Unigene0032023	-1.224403755	diphthine methyltransferase homolog isoform X1 (*Prunus mume*)
Unigene0038786	-1.223522782	WD repeat-containing protein 75 (*Juglans regia*)
Unigene0023327	-1.205421898	WD repeat-containing protein 3 (*Vitis vinifera*)
Unigene0037037	-1.185212251	WD repeat-containing protein 44 (*Nelumbo nucifera*)
Unigene0058895	-1.129144023	WD40 repeat (*Macleaya cordata*)
Unigene0008400	1.925910111	WD repeat-containing protein 25 (*Jatropha curcas*)

* Data were the mean value of three biological replicates.
